# Elevation of phosphate levels impairs skeletal myoblast differentiation

**DOI:** 10.1007/s00441-020-03254-1

**Published:** 2020-07-28

**Authors:** Adalbert Raimann, Alexander Dangl, Alireza Javanmardi, Susanne Greber-Platzer, Monika Egerbacher, Peter Pietschmann, Gabriele Haeusler

**Affiliations:** 1 Department of Pediatrics and Adolescent Medicine, Division of Pediatric Pulmonology, Allergology and Endocrinology, Comprehensive Center for Pediatric, Medical University of Vienna, Waehringer Guertel 18-20, 1090 Vienna, Austria; 2 Administrative Unit Veterinary Medicine, UMIT TIROL - Private University for Health Sciences,Medical Informatics and Technology GmbH, Wettermodifizierungszentrum 1, 6060 Hall in Tirol, Austria; 3 Department of Pathophysiology and Allergy Research, Center of Pathophysiology, Infectiology and Immunology, Medical University of Vienna, Wien, 1090 Austria

**Keywords:** Skeletal myoblast differentiation, Serum phosphate, Chronic kidney disease, Uremic sarcopenia, Hyperphosphatemia

## Abstract

Hyperphosphatemic conditions such as chronic kidney disease are associated with severe muscle wasting and impaired life quality. While regeneration of muscle tissue is known to be reliant on recruitment of myogenic progenitor cells, the effects of elevated phosphate loads on this process have not been investigated in detail so far. This study aims to clarify the direct effects of hyperphosphatemic conditions on skeletal myoblast differentiation in a murine in vitro model. C2C12 murine muscle progenitor cells were supplemented with phosphate concentrations resembling moderate to severe hyperphosphatemia (1.4–2.9 mmol/l). Phosphate-induced effects were quantified by RT-PCR and immunoblotting. Immunohistochemistry was performed to count nuclear positive cells under treatment. Cell viability and metabolic activity were assessed by XTT and BrdU incorporation assays. Inorganic phosphate directly induced ERK-phosphorylation in pre-differentiated C2C12 myoblast cells. While phosphate concentrations resembling the upper normal range significantly reduced Myogenin expression (− 22.5%, *p* = 0.015), severe hyperphosphatemic conditions further impaired differentiation (Myogenin − 61.0%, *p* < 0.0001; MyoD − 51.0%; *p* < 0.0001). Analogue effects were found on the protein level (Myogenin − 42.0%, *p* = 0.004; MyoD − 25.7%, *p* = 0.002). ERK inhibition strongly attenuated phosphate-induced effects on Myogenin expression (*p* = 0.002). Metabolic activity was unaffected by the treatments. Our data point to a phosphate-induced inhibition of myoblast differentiation without effects on cell viability. Serum phosphate levels as low as the upper normal serum range significantly impaired marker gene expression in vitro. Investigation of cellular effects of hyperphosphatemia may help to better define serum cutoffs and modify existing treatment approaches of phosphate binders, especially in patients at risk of sarcopenia.

**Electronic supplementary material:**

The online version of this article (10.1007/s00441-020-03254-1) contains supplementary material, which is available to authorized users.

## Introduction

Reduced motor performance and muscular dysfunction are major factors for morbidity and impairment of the quality of life in patients with chronic kidney disease (CKD) (Tenbrock et al. 2000; Eijsermans et al. 2004). The causes of CKD-associated myopathy include suppression of protein synthesis and impaired muscle growth by cardiovascular, metabolic, and/or hormonal abnormalities (Wang and Mitch 2014). The role of single factors such as elevated phosphate levels is difficult to define due to the multitude of possible confounders such as hyperparathyroidism, malnutrition, or chronic inflammation (de Souza et al. 2015). Hyperphosphatemia is known to be an important cause of morbidity in an increasing number of patients with CKD (Qunibi 2004; Sehgal et al. 2008; Hill et al. 2016). While the precise mechanisms are only partially understood, elevated phosphate levels have consistently been shown to be an independent risk factor for increased mortality in several conditions (Friedman 2005; Haider et al. 2015). In CKD, serum phosphate levels are known to increase in patients with glomerular filtration rates below 30% (Delmez and Slatopolsky 1992).

Impaired satellite cell recruitment has been identified as a key mechanism in the development and progression of CKD-associated muscle loss (Zhang et al. 2010). While regeneration of muscle tissue relies on recruitment of myogenic progenitor cells, the effects of high phosphate loads on this process have not been extensively investigated. In this study, we aimed to identify the role of elevated phosphate levels during C2C12 myoblast differentiation and early stages of differentiation to define the role of a clinically relevant and therapeutically targetable single factor.

## Materials and methods

### Research facility

Research facilities and infrastructure were provided by the Laboratory of Pediatric Endocrinology, Department of Pediatric and Adolescent Medicine, Medical University Vienna. All experiments were performed according to the official guideline for Good Scientific Practice. (http://www.meduniwien.ac.at/files/7/8/goodscientificpractice.pdf; 2019-07-11).

### Cell culture

Murine C2C12 (ATCC® CRL­1772™) myoblast cells (ATCC, Manassas, USA) were cultured according to the manufacturer’s instructions. C2C12 cells were harvested from three independent runs after 1, 2, and 6 days in differentiation media containing DMEM (Thermo Fisher Scientific, Vienna, Austria) and 2% horse serum (Thermo Fisher Scientific). Phosphate treatments were prepared by supplementation of a 67:33 mixture of 1M Na_2_HPO_4_ and 1M NaH_2_PO_4_ (Sigma-Aldrich, St. Louis, Missouri, USA) to the differentiation media. Treatment by either 0.5 mM phosphate or 2 mM phosphate resulted in effectively 1.42 mM and 2.92 mM phosphate. ERK1/2 inhibition was performed by supplementation of 10 μM U0126 (Merck, Darmstadt, Germany) to the differentiation media during 6 days of treatment.

### Gene expression analysis

TRI reagent (Sigma-Aldrich) was applied to adherent cells after washing with Hank’s media. RNA was isolated according to the manufacturer’s recommendations. The purity and amount of RNA was determined by measurement of an OD 260:280 ratio using a NanoDrop 2000 spectrophotometer (Thermo Fisher Scientific). About 500 ng of total RNA was transcribed using the iScript cDNA synthesis kit according to the manufacturer’s instructions (Bio-Rad, Hercules, California, USA).

PCR amplification was performed and monitored using a 7500 fast real-time PCR system (Applied Biosystems, California, USA). Master mix was based on SensiFAST SYBR No-ROX (Bioline Reagents Ltd., London, UK). The thermal cycling conditions comprised the initial steps at 50 °C for 2 min and at 95 °C for 10 min. Amplification of the cDNA products was performed with 40 PCR cycles, consisting of a denaturation step at 95 °C for 15 s and an extension step at 60 °C for 1 min. The final numeric value was calculated as the ratio of the target genes to the means of 18S RNA and beta-actin expression and expressed in arbitrary units using the delta-delta Ct method (Livak and Schmittgen 2001).

### Primers and probes for quantitative analyses

Primers and probes were designed using NCBI Primer Blast (Ye et al. 2012) to create oligo nucleotides with similar melting temperatures and minimal self-complementarities (Sup. Tab. 1). The probes were placed at the junction of two exons to avoid amplification of genomic DNA. The gene specificity of the primers and probes and the absence of DNA polymorphism were confirmed by BLASTN searches. Primers and probes were synthesized by GenXpress (Wiener Neudorf, Austria) or purchased as pre-prepared expression assay.

### Immunoblotting

C2C12 cells were lysed on day 6 in lysis buffer (SDS 10:TritonX100:0.5 mM EDTA 10:10:1) including protease inhibitors (Complete Mini Roche Diagnostics, Vienna, Austria) and phosphatase inhibitor sc-45045 (Santa Cruz Biotechnology). About 6 μg protein was used for blotting and incubated with P44/42 MAPK 1:1000 (Cell Signaling Technology, Frankfurt am Main, Germany), Phospho-p44/42 MAPK (1:1000, Cell Signaling Technology), Anti-Skeletal Myosin (1:1000, M 4276, Sigma-Aldrich, Darmstadt, Germany), or Anti-β-actin (sc-47778, Santa Cruz Biotechnology, Heidelberg, Germany) overnight at 4 °C. Membranes were washed thrice in TBST and incubated with anti-rabbit-IgG HRP-linked antibody no. 7074S or anti-mouse-IgG 7076S both 1:2000. Images were acquired by FUSION FX (Peqlab Biotechnologie GmbH, Erlangen, Germany). Quantifications were performed using ImageLite 4.0 (LI-COR Biosciences, Lincoln, Nebraska).

### Immunostaining

Before harvest at day 6 of incubation, adherent cells were washed with HANKS media (Thermo Fisher Scientific) and trypsinized. Pellets were prepared for immunostaining by centrifugation and fixation in 4% formaldehyde for 24 h. Two sections of each sample were mounted on slides with DPX (Fluka, Buchs, Switzerland), deparaffinized and rehydrated. Endogenous peroxidase activity was blocked by methanol (120 volumes) and hydrogen peroxide (1 volume). Protein block was performed with 1.5% goat serum (Dako/Agilent, Santa Clara, California). Sections were incubated with either mouse-anti-MyoD sc-32758 1:100 (Santa Cruz, Dallas, Texas) or rabbit-anti-Myogenin HPA038093 1:100 (Sigma-Aldrich) overnight at 4 °C. BrightVision Poly-HRP (ImmunoLogic, Duiven, Netherlands) was applied for 30 min according to the manufacturer’s instructions. Cell nuclei were counterstained with Mayer’s hematoxylin and slides mounted with DPX 141 (Fluka, Buchs, Switzerland). Positive nuclei and the total cell number were counted in 5 visual fields of 2 slides per sample by two independent, blinded observers.

### Proliferation and cellular viability

Cell proliferation was determined by a 5-bromo-20-deoxyuridine (BrdU) colorimetric assay (Roche Applied Science, Vienna, Austria) according to the manufacturer’s instruction. About 5000 cells were seeded in 96-well plates and differentiated in sextuplets with treatments identical to expressional experiments. BrdU was added at day 5 of differentiation and incubated for 24 h at 37 °C. Anti-BrdU-peroxidase (1:100) was added and incubated for 60 min.

Cellular viability was investigated with the EZ4U assay (Biomedica, Vienna, Austria) according to manufacturer’s instructions. About 20 μL of dye solution was added on day 6 of differentiation to each well followed by 60 min incubation at 37 °C.

The absorbance of both BrdU and EZ4U assays was measured at 450 nm with a reference wavelength of 620 nm using a VICTOR Microplate (PerkinElmer, Waltham, MA, USA) reader. Results are presented relative to non-treated controls (Fig. S1).

### Statistical analysis

All experiments were performed in ≥ 3 independent runs. One-way ANOVA followed by Tukey’s multiple comparison test was performed using GraphPad Prism version 8.00 for Windows (GraphPad Software, La Jolla, CA). Values of treated samples were normalized to non-treated controls. A *p* ≤ 0.05 was considered as statistically significant.

## Results

We analyzed the effects of inorganic phosphate on C2C12 myoblast cell differentiation on the gene and protein expression level. mRNA and nuclear protein expression were compared under euphosphatemic (0.92 mM), moderate (1.42 mM), and severe (2.92 mM) hyperphosphatemic conditions during differentiation. Further, ERK phosphorylation by inorganic phosphate, cellular proliferation rates, and metabolic activity under hyperphosphatemic conditions were analyzed.

### Gene expression

**Fig. 1:**
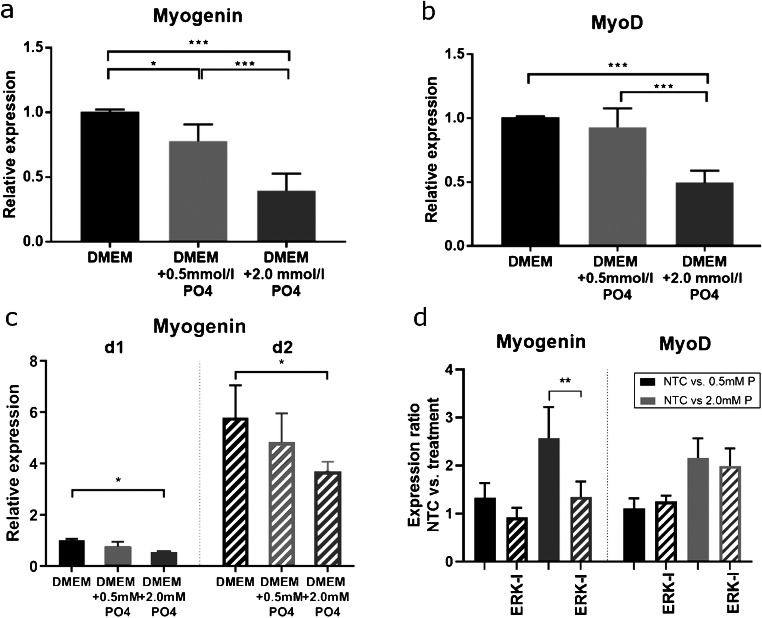
**a**, **b** mRNA expression profile of C2C12 myoblasts after differentiation in phosphate supplemented media**.** Increased phosphate concentrations significantly decreased marker gene expression after 6 days of treatment. Myogenin expression was reduced by 2.0 mM phosphate at day 1 (d1) and day 2 (d2) of treatment (**c**). ERK1/2 inhibition attenuated high-dosage phosphate effects on Myogenin expression as shown by NTC to treatment ratios (**d**). Values are shown as mean + SD (*p < 0.05; **p < 0.01;***p < 0.001)

Myogenin*,* a transcription factor essential for myoblast differentiation, was profoundly downregulated under elevated phosphate concentrations (Fig. 1a). On day 6 upon differentiation, moderate hyperphosphatemic conditions decreased Myogenin expression significantly (− 23%, *p* = 0.015); a more pronounced drop in mRNA transcription at higher phosphate concentrations was observed (− 61%, *p* < 0.0001). A similar phosphate concentration reduced Myogenin expression at earlier time points of differentiation (Fig. 1c; day 1–47%, *p* = 0.004; day 2–50%, *p* = 0.039), while effects of 0.5 mM phosphate treatment remained non-significant (Fig. 1c; day 1–26%, *p* = 0.09;day 2–20%, *p* = 0.24).

Expression of MyoD after 6 days of hyperphosphatemic conditions revealed similar patterns as observed for Myogenin (Fig. 1b): MyoD was significantly downregulated under severe hyperphosphatemic conditions (− 51%, *p* < 0.0001). Decreased MyoD expression for moderate phosphate elevation did not reach significance (− 8%, *p* = 0.481). ERK inhibition itself increased Myogenin expression (data not shown) and strongly attenuated high-dosage phosphate effects (*p* = 0.002) as compared with inhibitor-free media (Fig. 1d). Phosphate-induced changes of MyoD expression remained unaltered.

### Protein expression

**Fig. 2:**
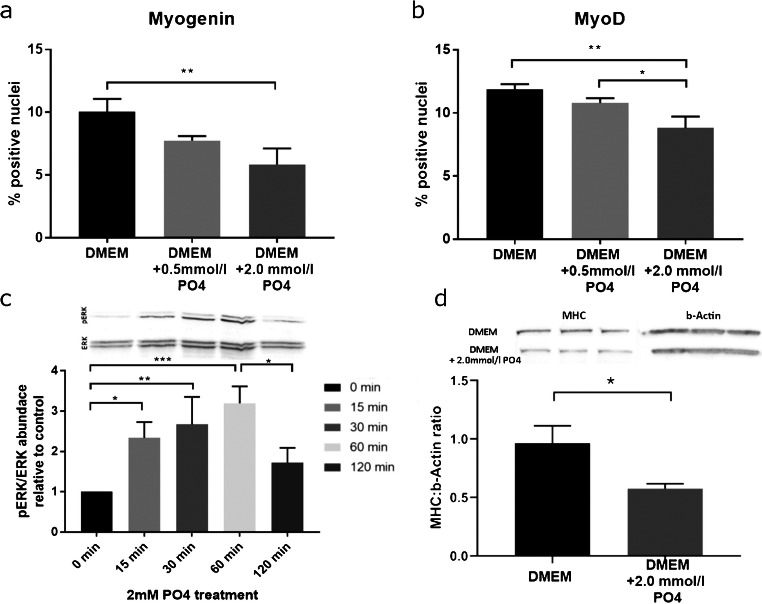
**a-c** Protein expression and cell signaling in phosphate-treated C2C12 myoblasts. Phosphate supplementation during differentiation significantly decreased the number of marker protein expressing nuclei (**a**,**b**). Phosphate directly induces ERK-phosphorylation 15 min after treatment in differentiated C2C12 cells (**c**). MHC protein expression was reduced after 6 days of 2.0 mM phosphate treatment as shown by MHC:b-Actin ratios (**d**). Values are shown as mean + SD (*p < 0.05; **p < 0.01; ***p < 0.001)

In order to confirm mRNA expression data on the protein level, we counted the Myogenin and MyoD-positive nuclei stained by immunohistochemistry (Fig. 2a, b). In line with the mRNA expression data, severe hyperphosphatemic conditions decreased the number of cells with nuclear expression of both Myogenin (− 42%, *p* = 0.004) and MyoD (− 26%, *p* = 0.002). Decreased counts for moderate elevation of phosphate did not reach significance (Myogenin − 22%, *p* = 0.058; MyoD − 9%, *p* = 0.165). Further, MHC protein levels were significantly reduced after 6 days of hyperphosphatemic conditions (Fig. 2d; − 38.9%, *p* = 0.025).

### ERK phosphorylation

ERK immunoblotting was performed to prove direct pathway activation by inorganic phosphate on myoblast cells (Fig. 2c). The pERK:ERK abundance ratio increased significantly 15 min after treatment (234%, *p* = 0.021), reaching a maximum after 60 min (320%, *p* = 0.001). At 120 min, ERK phosphorylation returned to non-significantly increased values compared with baseline (173%, *p* = 0.293).

### Proliferation and metabolic activity

Increases in media phosphate concentrations did not alter cell proliferation rates as measured by BrdU incorporation rates (*F* = 0.85, *p* = 0.479; Fig. S1a). Glucose metabolism as measured by tetrazolium reduction rates showed comparable results (Fig. S1b; *F* = 1.632 *p* = 0.272). Thus, the downregulation of gene and protein expression observed under hyperphosphatemic conditions were not associated with any impaired metabolic or proliferative activity of myoblasts.

## Discussion

Uremic sarcopenia is a major challenge in the care of patients with CKD. This study aimed to analyze the role of elevated phosphate levels in the process of skeletal myoblast differentiation to explain this clinically relevant symptom in CKD.

To our knowledge, this is the first report of direct activation of ERK phosphorylation by extracellular phosphate in cells of the skeletal myocyte lineage. Using the well-established C2C12 in vitro system, we used cell culture conditions to simulate phosphate concentrations just below the normal range to values, which have previously been found in severely hyperphosphatemic patients (Nolan and Qunibi 2005). Given that elevated serum phosphate even within the normal range is independently associated with cardiovascular mortality, it must be questioned if the normal range is an optimal target for patients at risk for myopathy (Tonelli et al. 2005). In our study, phosphate levels even in the upper normal range significantly decreased myoblast differentiation gene expression. The strongest suppression was observed for the expression of a key regulator of myoblast differentiation, Myogenin, which was in part rescued by ERK1/2 inhibition. Importantly, a significant reduction of Myogenin expression was observed after 1, 2, and 6 days of cultivation. While the effects on mRNA and protein level at day 6 could have been an effect on differentiated cells only, the observed reduction after 24 h and 48 h of cultivation rather point to an impairment of differentiation than to isolated, post-differentiational effects on Myogenin transcription. Interpreted in the context of CKD, most patients not yet on dialysis are affected by moderate elevations of serum phosphate as creatinine clearance declines below 40 ml/min although phosphate levels often remain within the upper normal range (Kestenbaum et al. 2005). Our data point to a yet underestimated sensitivity of skeletal muscle precursors towards alterations in phosphate concentrations. Early increases in serum phosphate could thus represent a first hit for the development of uremic sarcopenia, impairing damage repair, tissue regeneration, and resistance against toxic metabolites by impairing precursor recruitment.

A limitation of our study is the myoblast in vitro model system, allowing conclusion on myoblast differentiation only, without information on mature muscle fiber function. Nevertheless, myoblast differentiation after recruitment of satellite cells is a key mechanism for damage repair and was shown to be affected in several CKD animal model studies (Wang et al. 2009; Zhang et al. 2010). Any possible damaging effect on the skeletal muscle might be potentiated by even marginally impaired differentiation of these myoblasts to regenerate muscle tissue. While our data do not allow to draw conclusions on the process of recruitment itself, the amount of decrease in MyoD and Myogenin expression observed in CKD mice corresponds well to our data from cells treated with elevated phosphate concentrations. Importantly, high normal phosphate conditions in our model already resemble the values observed in myoblasts of CKD mice, emphasizing the relevance of the observed effect size. Given the treatment options for hyperphosphatemia, identification of phosphate as a possible risk factor for the vulnerable system of skeletal muscle regeneration may present an attractive target for further investigations. Revealing and identifying cellular responses to excess phosphate may help to set serum phosphate cutoffs and modify existing treatment approaches of phosphate binders, especially in patients at risk for myopathy.

## Electronic supplementary material

Supplementary Figure S1 a, b**-** Effects of phosphate treatments on C2C12 myoblast metabolism. Phosphate supplementation did neither alter proliferation rate (S1a) nor metabolic activity (S1b) of C2C12 myoblasts). Values are shown as mean + SD Figure S2: IHC stainings of C2C12 myoblast C2C12 myoblasts were harvested and stained for Myogenin and MyoD expression. Representative sections are shown for NTC, 0.5mM and 2.0mM phosphate supplementation. Clearly positive nuclei were divided by the total number of cells per slide to calculate the percentage of expressing cells (PNG 11796 kb)

High resolution image (TIFF 214 kb)

Supplementary Table 1 Primer *sequences* for quantitative RT-PCR (PDF 89 kb)
