# Supplementary figures and images for: Elevation of phosphate levels impairs skeletal myoblast differentiation
Source: Cell Tissue Res. 2020 Jul 28;382(2):427–32. doi: 10.1007/s00441-020-03254-1 (PMC7584532; doi:10.1007/s00441-020-03254-1)

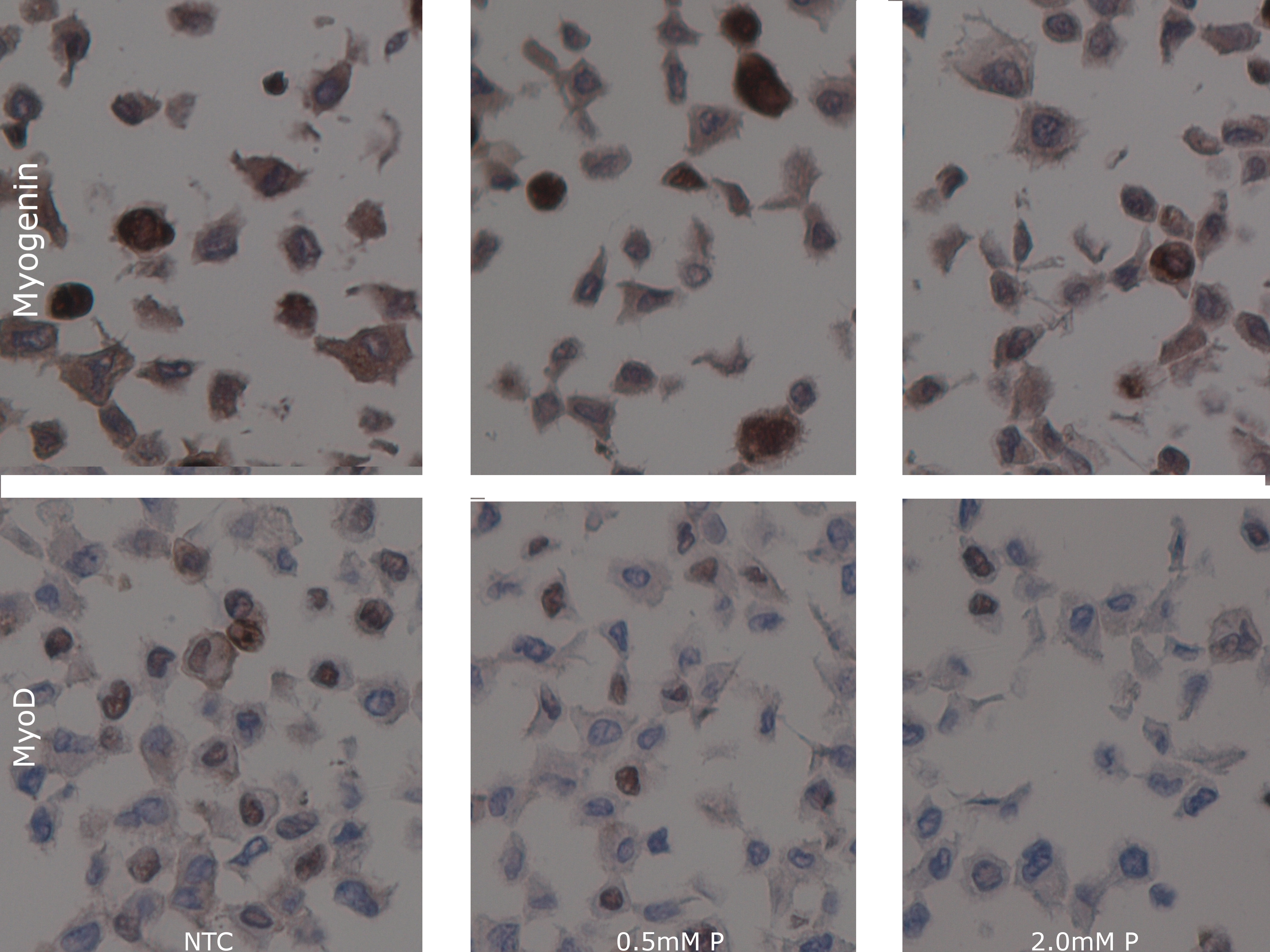

Supplement: Supplementary file 1 — a, b- Effects of phosphate treatments on C2C12 myoblast metabolism. Phosphate supplementation did neither alter proliferation rate (S1a) nor metabolic activity (S1b) of C2C12 myoblasts). Values are shown as mean + SD Figure S2: IHC stainings of C2C12 myoblast C2C12 myoblasts were harvested and stained for Myogenin and MyoD expression. Representative sections are shown for NTC, 0.5mM and 2.0mM phosphate supplementation. Clearly positive nuclei were divided by the total number of cells per slide to calculate the percentage of expressing cells (PNG 11796 kb) [file 441_2020_3254_MOESM2_ESM.png]

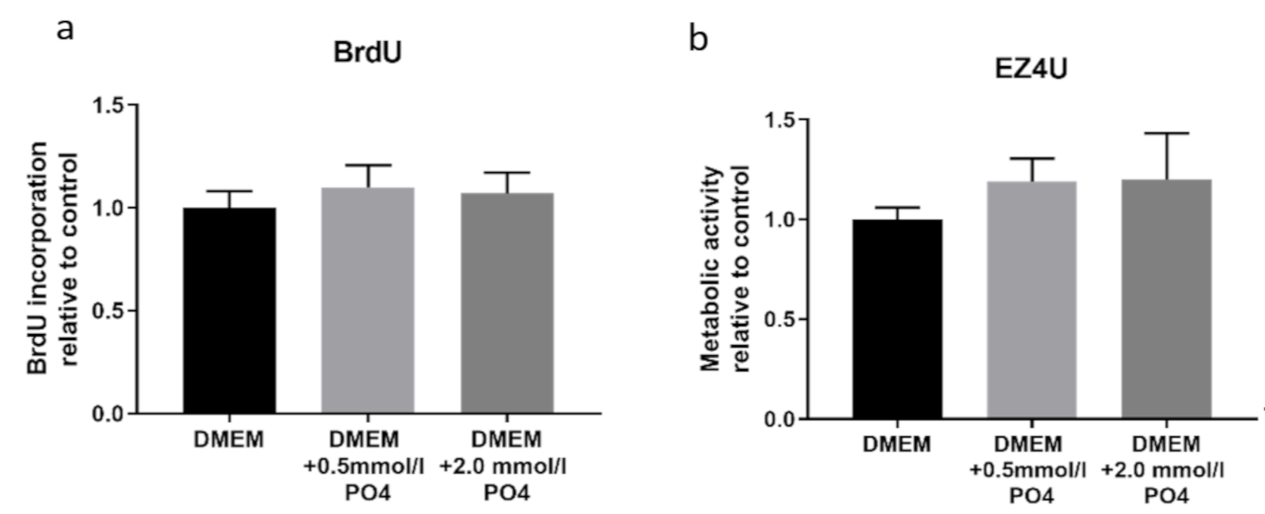

Supplement: Supplementary file 2 — High resolution image (TIFF 214 kb) [file 441_2020_3254_MOESM1_ESM.tiff]
