# Supplementary material for: Elevation of phosphate levels impairs skeletal myoblast differentiation
Source: Cell Tissue Res. 2020 Jul 28;382(2):427–32. doi: 10.1007/s00441-020-03254-1 (PMC7584532; doi:10.1007/s00441-020-03254-1)
Supplement: Supplementary file 3 — Primer sequences for quantitative RT-PCR (PDF 89 kb) [file 441_2020_3254_MOESM3_ESM.pdf]

Gene		sequence / manufacturer
Myogenin	forward	CCAGCGGCTGCCTAAAGTGG
	reverse	GCCGCGAGCAAATGATCTCCTG
MyoD	forward	TGATGGCATGATGGATTACAGCGG
	reverse	GGGTTCCCTGTTCTGTGTCGC
b-Actin	forward	CAGCAAGCAGGAGTACGATGA
	reverse	GGTGTAAAACGCAGCTCAGTA
18S		TaqMan® Gene Expression Assay
		(Thermo Fisher Scientific)
